# COVID-19-Related Mental Health Burdens: Impact of Educational Level and Relationship Status Among Low-Income Earners of Western Uganda

**DOI:** 10.3389/fpubh.2021.739270

**Published:** 2021-11-18

**Authors:** Ann Monima Lemuel, Ibe Michael Usman, Keneth Iceland Kasozi, Saad Alghamdi, Eric Osamudiamwen Aigbogun, Victor Archibong, Robinson Ssebuufu, Annet Kabanyoro, Josiah Eseoghene Ifie, Dominic Terkimbi Swase, Fred Ssempijja, John Tabakwot Ayuba, Kevin Matama, Hope Onohuean, Stellamaris Kembabazi, Rachael Henry, Said Odoma, Helen Yusuf, Adam Moyosore Afodun, Hamza M. Assaggaf, Emmanuel Kairania, Akhmed Aslam, Owoisinke Okon, Gaber El-Saber Batiha, Susan Christina Welburn

**Affiliations:** ^1^Faculty of Biomedical Sciences, Kampala International University Western Campus, Bushenyi, Uganda; ^2^Infection Medicine, Deanery of Biomedical Sciences, College of Medicine and Veterinary Medicine, The University of Edinburgh, Edinburgh, United Kingdom; ^3^School of Medicine, Kabale University, Kabale, Uganda; ^4^Department of Laboratory Medicine, Faculty of Applied Medical Sciences, Umm Al-Qura University, Makkah, Saudi Arabia; ^5^Department of Public Health Science, Faculty of Science and Technology, Cavendish University, Kampala, Uganda; ^6^Uganda Medical and Dental Practitioners' Council, Kampala, Uganda; ^7^School of Nursing, Kampala International University Teaching Hospital, Bushenyi, Uganda; ^8^Department of Pharmacology and Toxicology, School of Pharmacy, Kampala International University Western Campus, Kampala, Uganda; ^9^Biopharmaceutics Unit, Department of Pharmacology and Toxicology, School of Pharmacy, Kampala International University, Kampala, Uganda; ^10^Department of Human Anatomy, College of Medicine and Health Science, Ahmadu Bello University, Zaria, Nigeria; ^11^Department of Pharmacology and Toxicology, International University, Kampala, Uganda; ^12^Department of Pharmacology, College of Health Sciences, Kogi State University, Anyigba, Nigeria; ^13^Department of Anatomy and Cell Biology, Faculty of Health Sciences, Busitema University, Tororo, Uganda; ^14^Department of Public Health, University of Calabar, Calabar, Nigeria; ^15^Department of Pharmacology and Therapeutics, Faculty of Veterinary Medicine, Damanhour University, Damanhour, Egypt; ^16^Zhejiang University-University of Edinburgh Institute, Zhejiang University School of Medicine, Zhejiang University, Haining, China

**Keywords:** mental healthcare, awareness, relationship status, educational level, COVID-19, low-income earners, Western Uganda

## Abstract

**Objective:** The study aimed to investigate the relationship between mental health with the level of education, relationship status, and awareness on mental health among low-income earners in Western Uganda.

**Methods:** This was a cross-sectional descriptive study carried out among 253 participants. Anxiety, anger, and depression were assessed using a modified generalized anxiety disorder (GAD-7), Spielberger's State-Trait Anger Expression Inventory-2, and Beck Depression Inventory item tools, respectively.

**Results:** The majority of our respondents were male (*n* = 150/253, 59.3), had a secondary level of education (104/253, 41.1), and were single (137/253, 54.2). No formal education and primary education (*r*^2^ = 47.4% and 6.4%, respectively) had a negative correlation with awareness of mental health care. In addition, no formal education had a positive correlation with anger and depression (*r*^2^ = 1.9% and 0.3%, respectively). Singleness in this study had a negative correlation with awareness of mental health care, anger, and depression (*r*^2^ = 1.9, 0.8, and 0.3%, respectively), and a positive correlation with anxiety (*r*^2^ = 3.9%).

**Conclusion:** It is evident that education and relationship status influenced awareness on mental health care and mental health state among low-income earners in Western Uganda during the first COVID-19 lockdown. Therefore, policymakers should strengthen social transformation through the proper engagement of low-income earners in this COVID-19 era.

## Introduction

The global pandemic has entered its second year since it was first reported among patients in Wuhan city of China by late 2019 (1). The virus, initially named 2019 nCoV by the World Health Organization (WHO) was later renamed as severe acute respiratory syndrome coronavirus-2 (SARS-CoV-2) by the International Committee on Taxonomy of Viruses (2). It was identified by the Chinese Center for Disease Control and Prevention as the causative agent of pneumonia-like infection presenting with symptoms of a cough and high fever. While respiratory distress syndrome was initially reported as the primary pathology in patients with COVID-19, several systems have since been implicated, including the nervous system (3). As of August 22, 2021, over 211 million confirmed cases and 4.4 million death were reported globally (4). In Africa, 5,459,743 confirmed cases and 130,407 deaths were reported in WHO's coronavirus disease weekly epidemiological update (2021).

While the extent of the damage caused by the COVID-19 pandemic in Africa remains unclear, its impact on the socio-economic and mental health status is evident. A study among pediatric health care workers revealed high levels of depression and anxiety in the study population during the pandemic in China (1). The impact of the pandemic on the physical human health and social and political patterns create positive feedback that characteristically and indirectly inhibits the social, economic, and political wellbeing of communities, societies, and nations. As the pandemic progressed, there were projections that the mental health burden would increase as measures taken by the African governments to mitigate the spread of the SARS-COV-2 virus, such as the shelter-in-place directive, banning public gatherings, closure of schools, and social distancing, which have a great impact on the lives of people in low-income countries (5); with low-income earners, likely to be more affected (6, 7). Unfortunately, this particular group accounts for a larger proportion of the Ugandan population (8). Low-income earners are working people whose income falls below a given poverty line, which for Uganda this is less than $1 USD per day (9). This set of people spend at least 27 weeks in a year working or looking for gainful employment but remain under the poverty threshold (9). Individuals and families who are in lower economic classes have limited access to information, resources, and services that can help them to save and accumulate assets over time and as such depend on day-to-day business to make ends meet (10).

Public health emergencies can have psychological and economic effects on people at different levels in society, expressed as anxiety, fear, and worry (11). The pandemic combined with social distancing and the associated movement restriction created very stressful situations, resulting in elevated fear, irritability, depression, or anxiety (12). This situation is in most cases compounded by some sociodemographic factors, such as relationship status and level of education (12). Previous studies have found a strong association between separation or divorce and an increased risk of depression (13). Effective communication, support, and responsiveness are some factors that can help a relationship thrive during times of crisis and also enable partners to maintain stable mental health status.

Mental health care awareness has attracted widespread attention over the years. Higher levels of education are associated with increased access to a wide range of information, higher employment accessibility, and improved health and promote inter-personal and intra-personal wellbeing (14). Lopez et al. (15) stated that individuals who have attained some level of college education have a significantly higher knowledge of mental health care than their counterparts with low levels of education.

The study aimed to investigate the relationship between mental health with the level of education, relationship status, and awareness on mental health among low-income earners in Western Uganda. These data would be of value in guiding policymakers to evolve strategies focused on social transformation guided by effective mental health education and supporting low-income earners toward resilience during public health emergencies such as the COVID-19 pandemic.

## Materials and Methods

### Study Setting and Design

This was a cross-sectional descriptive study, involving 253 respondents (150 men and 103 women), aimed at investigating the implications of education level and relationship status on the level of awareness on mental health among low-income earners in Western Uganda, during the lockdown in the month of May 2020. This was during the initial stages of the first COVID-19 National Total Lockdown in Uganda, and this period was stressful to most of the vulnerable communities that we visited.

### Study Participants

The present study deployed a random sampling technique, among low-income earners between 18 and 65 years of age living and/or working within Ishaka municipality in the Bushenyi district of Western Uganda who gave their consent to be part of the study. Those outside the age required for the study were excluded from the study.

The study covered a total of 10 market areas in Bushenyi; Kizinda open market, Kizinda market area, African village market, Lagos market area, Bassajja market area, Ishaka open market, Kampala International University (KIU) market, Abuja market area, Ishaka business area, and KIU open market, respondents approached at random in the designated market areas.

For the purpose of this study, low-income earners were taxi drivers, market vendors, barbers, hairdressers, photocopying center attendees, etc. The study targeted this set of people since they were defined as vulnerable persons and COVID-19 restrictions affected their enterprises the most leading to increased incidents of poor mental health outcomes through increased isolation and job loss since they depend mostly on day-to-day work to make a living (16).

### Sample Size Determination

Since the study population is infinite, the study adopted the sample size necessary for estimating a population proportion of a small infinite population with (1-α) 100% confidence and error no larger than *e* (17)


$$\begin{array}{l} m=\frac{Z_{\frac{1}{2}\beta }^{2}\;p\left(1-p\right)}{e^{2}} \end{array}$$

*m* = is the sample size necessary for estimating the proportion *p* for a small infinite population, and *n* = correction to represent a finite population.

Let α = 5, therefore *e* = 0.05


$$\begin{array}{l} Z_{\frac{1}{2}\beta }\;=1.96 \end{array}$$

Where *p* = the proportion of low-income earners in Bushenyi.

However, available data only represents the poverty index of Bushenyi of 29.5% as in 2006 (18).

*p* = assumption that proportion of low-income earners will be around 50% of poverty index = 0.295 × 0.5 = 0.1475.

Therefore,


$$\begin{array}{ll} p & =0.1475.\\ m & =\frac{1.96^{2}\;\times \;0.1475\left(1\;-\;0.1475\right)}{0.05^{2}}\\ m & =\frac{0.48305719}{0.05^{2}}=193.22=193 \end{array}$$

The sample size for low-income earners was 193, and the researcher assumes an attrition rate of 10% (19); therefore, the workable sample size was 212. In the end, 280 potential respondents were approached, out of whom 27 of them declined. Therefore, the sample size was 253.

### Measurements and Data Collection Methods

A closed-ended questionnaire was used to collect data from the respondents who met the inclusion criteria for the study. The questionnaire had questions covering five different areas of the study; sociodemographic (gender, educational status, and relationship status), awareness on mental health care, anxiety, anger, and depression. Awareness of mental health care was assessed using the following questions: Do you know what mental health care is? Do you know any facility in Uganda where mental health care is provided? Is there a place in your locality where mental health care is provided? Do you have a pre-existing mental health challenge? (Yes or No). Which of the following mental health challenges apply to you? (Anxiety, Depression, Paranoia, Anger issues, Others, Not available). Anxiety was assessed using a modified generalized anxiety disorder (GAD-7) item tool (20). Anger was assessed using a modified Spielberger's State-Trait Anger Expression Inventory-2 (STAXI-2) (21). Depression was assessed using a modified Beck Depression Inventory (BDI) (22). Responses from the different components of the questionnaire were assigned scores.

Trained interviewers were recruited for the designated study locations, each of the interviewers had a Google format of the questionnaire on their devices, in order to minimize the exchange of writing material associated with the use of printed copies of the questionnaire. The interviewers also had copies of the consent form and introductory letter; the latter was to facilitate community entry, while the former was to facilitate the recording of consent by the respondents by appending their signatures.

The internal consistency for the different segments of the questionnaire (awareness on mental health care, GAD-7, STAXI-2, and BDI), Cronbach's α = 0.85, 0.79, 0.84, and 0.75, respectively, was determined by the data analyst in the research group.

### Data Management and Organization

**Figure 1:**
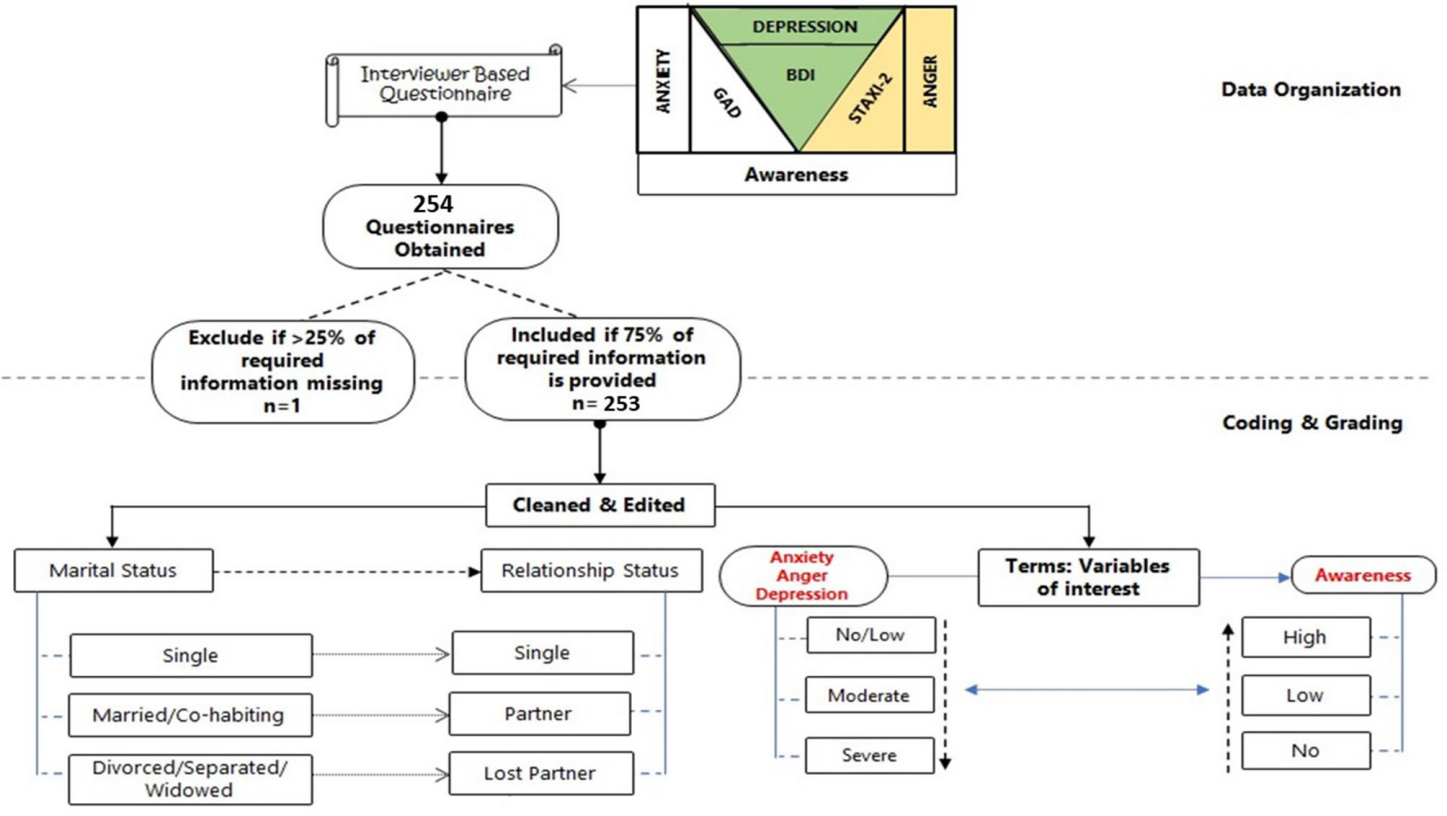
Data management (organization, coding, and grading).

The information entered *via* the Google form was retrieved in Microsoft Excel (2016) spreadsheet. Scores were assigned to each option as follows: Mental Health Care Awareness (Q5–Q10): Numerical values–mental health awareness [positive response = 1, negative response = 0]. Modified GAD assessment of anxiety (q11–q16): numerical values–multiple response [for each option selected = 1, indifferent = 0]. Modified STAXI-2 assessment for anger (q17–q23): numerical values–multiple response [for each option selected = 1, indifferent = 0]. Modified BDI assessment for depression (Q24–Q30): Numerical values–single graded response [highest grade of 3, indifferent = 0]. However, the data collected were assessed for completeness, and responses failing to meet the 75% cut-off (on all valid questions) were excluded (Figure 1).

For questions on awareness level, every positive response was assigned (1) and every negative response was assigned (0). The scores of the multiple options for the modified GAD and STAXI-2 were obtained by assigning one (1) mark per response. BDI had four (4) options graded as 3, 2, 1, and 0 (for indifferent). For specific graded questions (Yes, sometimes, or No), scores; 2, 1, 0 were assigned and all questions in this form were cumulated (per row), and the averages were obtained by summing all scores (qt) and dividing the number of questions (*n*) for each section. On the other hand, the obtained scores for each individual in different segments were then converted to percentages, to get the mean percentage scores for awareness, anxiety, anger, and depression.

### Data Analysis

The data were transferred to Graphpad Prism version 6 and Minitab 18.1 (Minitab, Inc. 2017) for analysis. The relationship between educational status and relationship status with awareness, anxiety, anger, and depression level were examined using Spearman's Rho correlation, then, all significant correlates were regressed using a system-assisted regression model. All analyses were performed at a 95% confidence level and *p* < 0.05 were taken to be significant.

## Results

**Figure 2:**
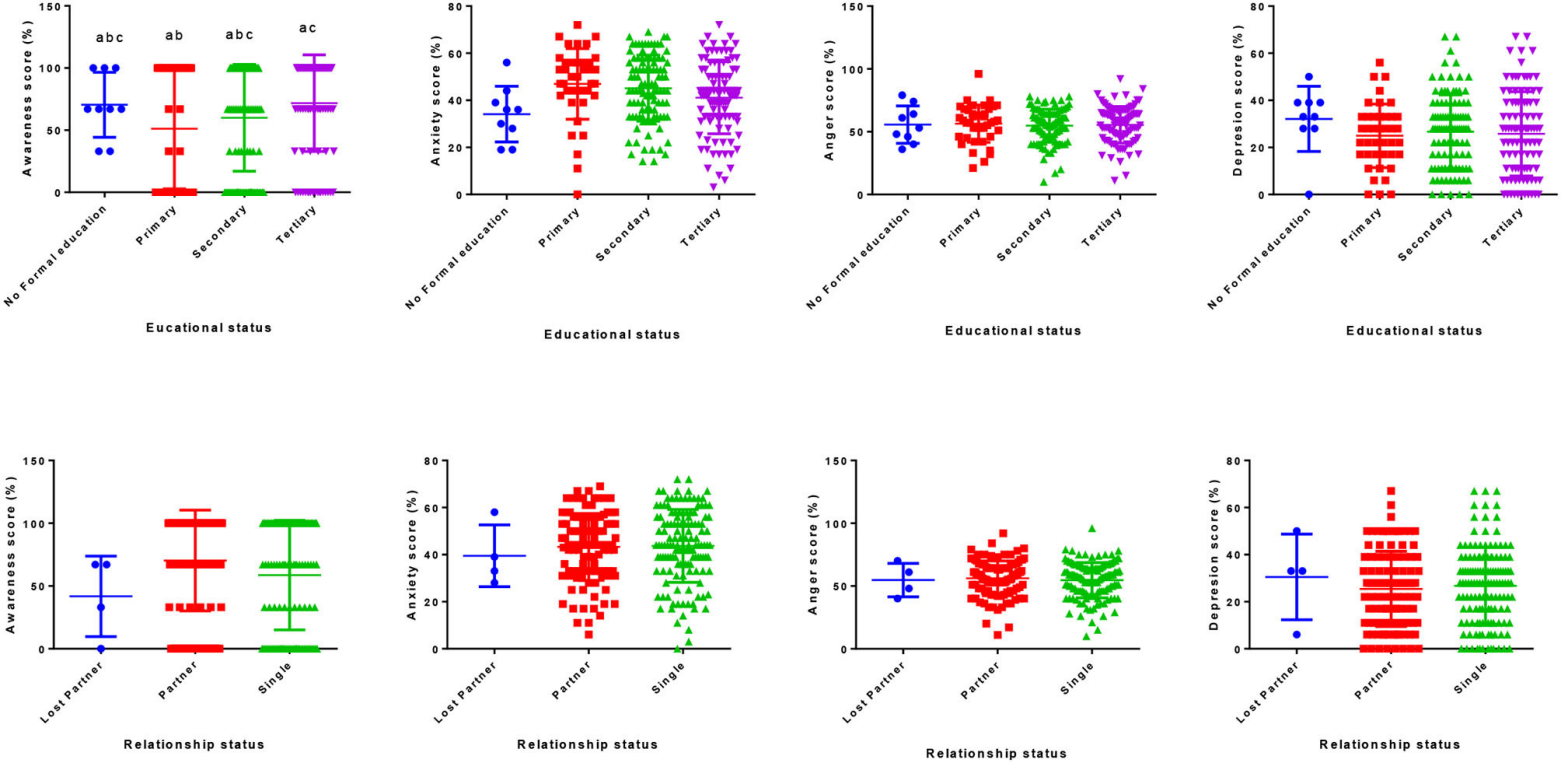
Score for awareness, anxiety, anger, and depression among various education and relationship statuses.

The study recruited 253 respondents, out of whom (*n* = 39) 3.6% had no formal education (*n* = 41) 16.2% had attained primary level of education (*n* = 104) 41.1% had attained a secondary level of education, and (*n* = 99) 39.1% had tertiary level of education (post-secondary or high school experience, diploma, and bachelor) (Table 1). The results showed that those with no formal education, primary, secondary, and tertiary level of education had percentage mean scores of 70.44, 51.22, 59.97, and 71.76%, respectively, for awareness. Respondents with primary education level and single individuals had a higher mean score (47.0 and 43.7%, respectively) for anxiety. Respondents with primary education level and those living with partners had higher mean scores (56.4 and 56.2%, respectively) for anger. Respondents with no formal education and those who had lost partners had higher mean scores (32.1 and 30.5%, respectively) for depression (Figure 2).

**Table 1:** Demographics features of the study population.

**Characteristics**	**Categories**	**Frequency (%)**	**95% CI**
Gender	Male	150 (59.3)	53.1–65.2
	Female	103 (40.7)	34.8–46.9
Education level	No formal education	9 (3.6)	1.8–6.4
	Primary	41 (16.2)	12.1–21.1
	Secondary	104 (41.1)	35.2–47.3
	Tertiary	99 (39.1)	33.3–45.3
Relationship status	Lost partner	4 (1.6)	0.5–3.8
	Partners	112 (44.2)	38.2–50.4
	Single	137 (54.2)	48.0–60.2

*Sample size = 253*.

Based on relationship status, respondents who were in the lost-partner group made up (*n* = 4) 1.6% of the sample population (*n* = 112) 44.2% were respondents who had partners, and (*n* = 137) 54.2% were respondents who were single. A breakdown of the statistics showed that respondents who had partners had a score of 70.28% for awareness, respondents who were single had a score of 58.66%, while respondents who had lost partners had a score of 41.75% for awareness.

**Figure 3:**
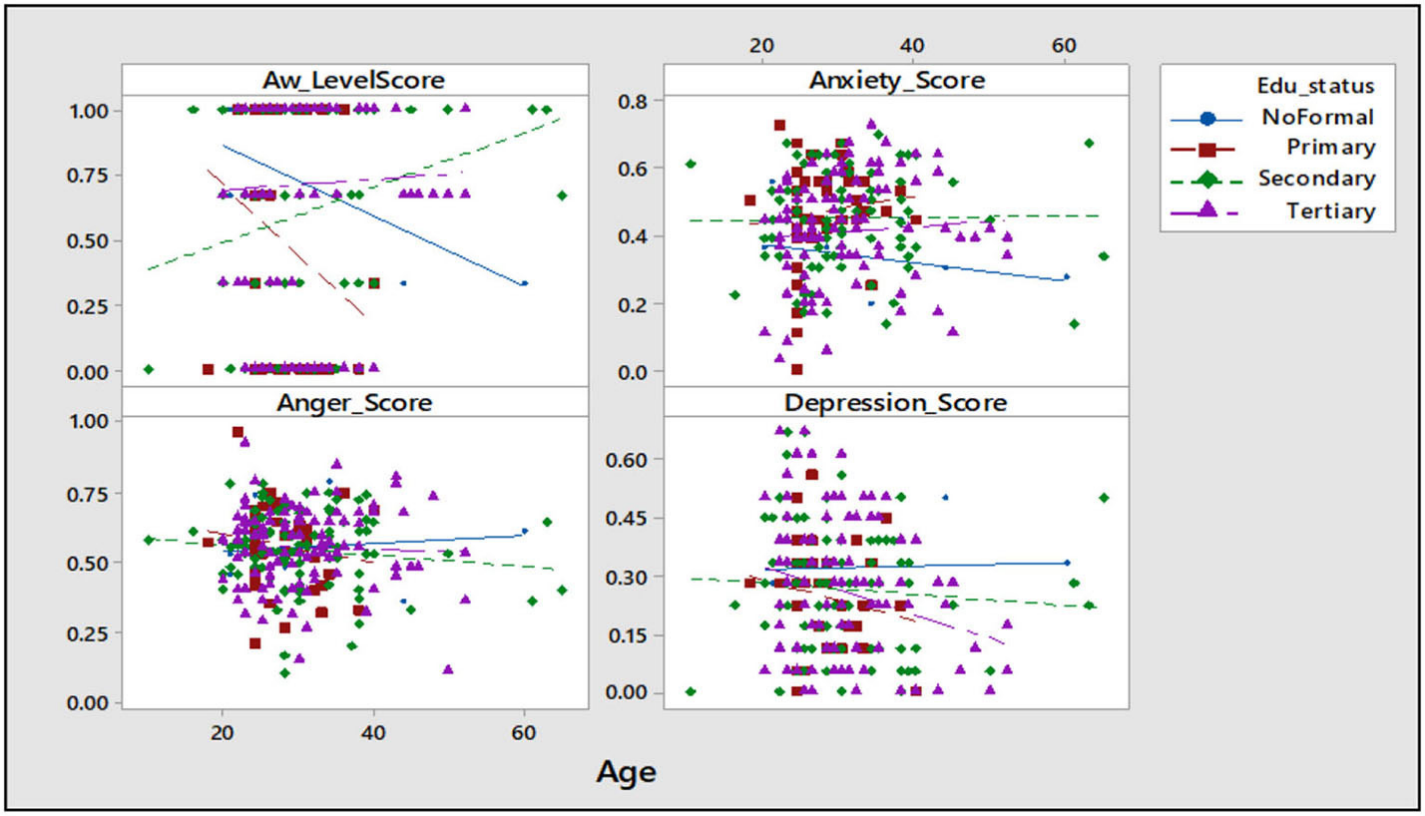
Scatter plot of awareness, anxiety, anger, and depression *vs*. educational status. AW, awareness; Sample size = 253.

**Figure 4:**
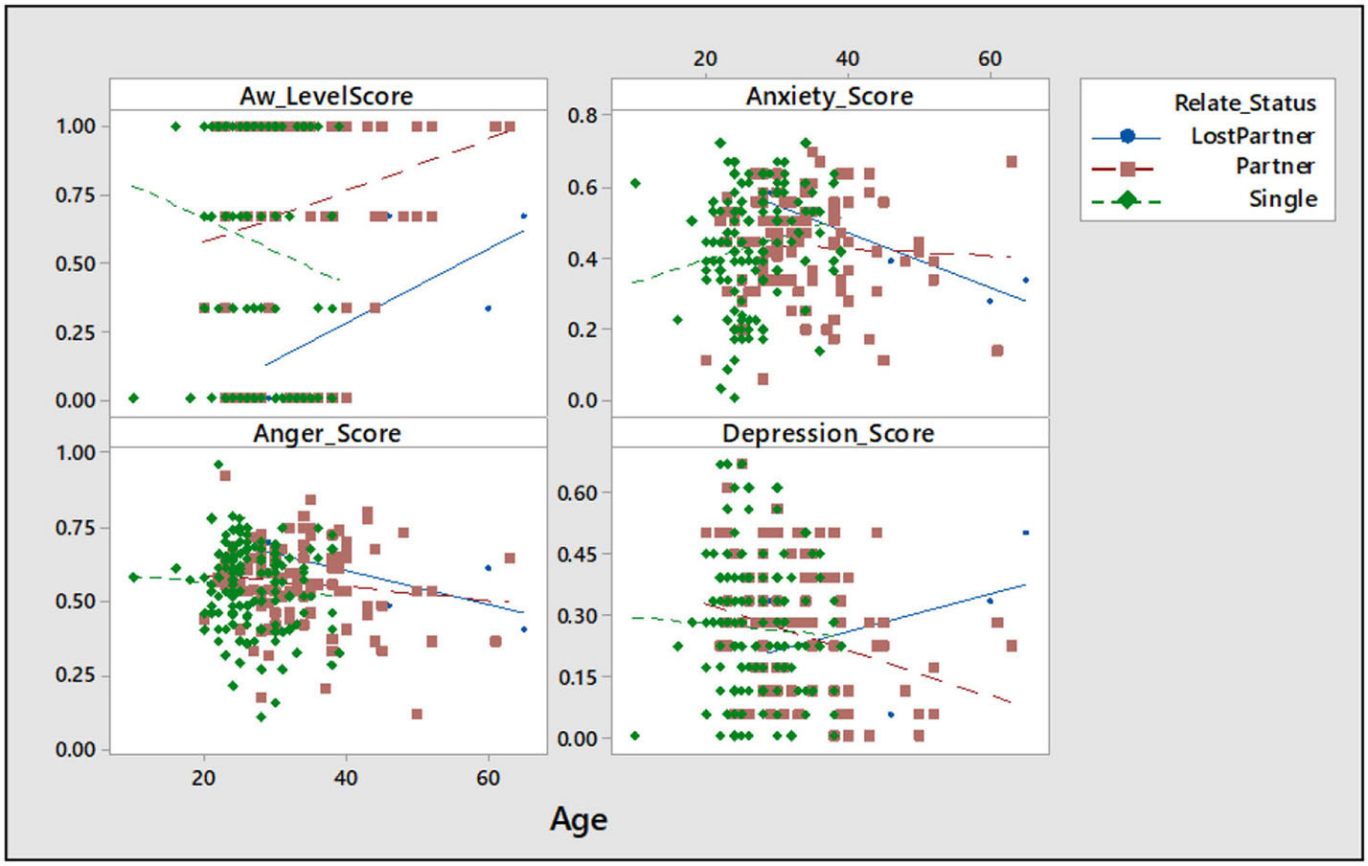
Scatter plot of awareness, anxiety, anger, and depression vs. relationship status. AW, awareness; Sample size = 253.

Educational status was observed to influence depression as age increases. The individuals with no formal education and primary education were observed to have a negative correlation (*r*^2^ = 47.4 and 6.4%, respectively) with awareness on mental health care, and the no formal education had a positive correlation with anger and depression (*r*^2^ = 1.9 and 0.3%, respectively) (Table 2; Figure 3). In comparing the age-associated chances across the relationship status group, it was observed that the lost-partner group had older individuals and were more aware, but also more depressed as age increased when compared to the single groups, which have a negative correlation (*r*^2^ = 1.9%) with awareness on mental health care, positive correlation (*r*^2^ = 3.9%) with anxiety, and negative correlation with anger and depression (*r*^2^ = 0.8 and 0.3%, respectively) (Table 2; Figure 4).

**Table 2:** Relationship (accuracy [*R*^2^] and direction) of age with awareness, anxiety, anger, and depression; stratified by sociodemographic characteristics.

**Variables**		**Age (accuracy: % [relationship])**			
		**Awareness**	**Anxiety**	**Anger**	**Depression**
Educational status	No formal	47.4% (–ve)	10.1% (–ve)	1.9% (+ve)	0.3% (+ve)
	Primary	6.4% (–ve)	1.3% (+ve)	2.5% (–ve)	3.0% (–ve)
	Secondary	4.5% (+ve)	0.0% (+ve)	1.6% (–ve)	0.6% (–ve)
	Tertiary	0.1% (+ve)	0.0% (+ve)	0.2% (–ve)	6.4% (–ve)
Relationship status	Lost-partner	47.0% (+ve)	87.6% (–ve)	48.9% (–ve)	16.4% (+ve)
	Partner	3.7% (+ve)	0.3% (–ve)	1.5% (–ve)	8.1% (–ve)
	Single	1.9% (–ve)	3.9% (+ve)	0.8% (–ve)	0.3% (–ve)

*Sample size = 253*.

## Discussion

The study was aimed at examining the relationship between mental health state and level of education, and relationship status among low-income earners in Uganda. Respondents with no formal education and those with tertiary level of education were more aware of mental health care services. Anxiety score was higher among respondents with a primary and secondary level of education compared with those with no formal education and tertiary level of education. Anger scores increased with age in respondents who had no formal education and respondents who had primary education level when compared to those who had secondary and tertiary levels of education demonstrating the relevance and urgency of the current study in a developing country. In this study, anger was higher in respondents who had partners when compared to those who lost partners and those who were single.

A low level of education among those without formal education and those who stopped in primary school accounts for the increased anger during the COVID-19 lockdown in Western Uganda. Boylan and Ryff (19) reported that those with higher educational qualifications tend to manage anger better than those with lower educational qualifications, and this was in agreement with our findings. Although anger seems to be a normal part of human existence and coping mechanism for stress (23), it could constitute a serious health problem. Especially, when motivated by events such as job loss and isolation associated with the pandemic (24, 25).

The observed high score for awareness among those with tertiary level of education in relation to those with primary and secondary levels of education suggests that education level significantly contributed to mental health awareness with persons more educated being more informed. The reported elevated level of awareness of mental health care services among those with tertiary level of education may be linked with the increase in knowledge-seeking attitude with an increase in the level of education. Feinstein et al. (26) reported that people with a higher level of education tend to seek more knowledge or information, thus supporting arguments presented in this study. In addition, they tend also to take advantage of available health care services compared to those with lower education levels (26).

The present study showed that respondents who had partners had more awareness of mental health care when compared to other groups of relationship status. The increase in awareness of mental health care was observed in the respondents who had partners who helped them handle stress effectively. Awareness of mental health care service is a cardinal step if we must experience a surge in uptake, especially in a trying time like this among vulnerable groups, like our study population (15, 27, 28).

The present study showed that anxiety increased among low-income earners who were single individuals compared to those with partners (married) and those who had lost their partners (separated/widowed). Single people experienced more anxiety during the COVID-19 lockdown in Western Uganda compared to those who are living with a partner or had once been in a marital relationship, contrary to popular opinion since one could assume that a single person has less household liabilities. The study affirms that intimate relationships tend to provide an avenue for people to express and manage personal distress, which may help alleviate anxiety (29). Marriage is associated with psychological benefits that make the partners happier and healthier (30–32). In marital relationships, the wives were more vulnerable to anxiety than husbands and those wives often perceived that their husbands were the ones who alleviated or appeased their anxiety (29). A study on “Singlism” observed that participants recruited in the study described married people as happy, fulfilled, stable, reliable, kind, giving, and loving, while on the other hand, single people were described as unsuccessful and ugly (33), thus portraying the pressures and expectations on single people which may contribute to anxiety over their relationship status, especially in a lockdown. In addition, single people have been reported to negatively evaluate other singles as being more at risk and unstable (34).

People often find it much easier to express their displeasure in the form of anger in the presence of their partners; this is most common among older couples (35). Anger expression comes with a lot of relief for most people; therefore, this makes them less likely to express symptoms of depression (36). Emotional attraction to one's partner decreases as a relationship grows older, therefore, it is not out of place to register dissatisfaction (35). Globally the lockdown was associated with job loss, a decline in possible income, inability to provide for one's partner, and disruption in normal life activity (37, 38). These events have been associated with the feeling of hopelessness, which is the major cause of anger. This was the case in a study conducted by Gordeeva et al. (39) among university students in Moscow during the lockdown. They reported a significant correlation between anger and the feeling of hopelessness (39).

This study also showed an increase in depression score with an increase in age among those who lost a partner. Separation or divorce is associated with an increased risk of depression, although there are inconsistencies as to whether the increase is higher among women or men (12, 40, 41). Being previously married (lost partner/separated) was associated with increased risk of depressive disorders; thereby supporting the fact that getting married is in most cases associated with the lowered risk of depression and alcohol use (42), and maybe the reasons behind the increase in the depression score in separated groups when compared to partner and singles group in our study. This may be a confirmation of the fact that the separated group experienced more depression because anger is one of the ways most people express depression (43).

There is no information on the mental health status and awareness of mental health care services among our study population prior to the pandemic. This would have enabled us possibly evaluate the impact of the nationwide lockdown on the mental health status and awareness of mental health care services among our study population, thereby constituting a limitation in the present studies.

## Conclusions

Educational level and relationship status had an influence on the awareness of mental health care and mental health state among low-income earners in Western Uganda during the COVID-19 lockdown. Our study showed that respondents who had no formal education and those who lost their partners were more affected by the COVID-19 lockdown in Western Uganda, which was why they had higher scores for depression when compared to other groups. The current finding could help in coming up with follow-up interventional programs capable of adequately addressing the possible impact of the nationwide lockdown among low-income earners in Western Uganda, with adequate attention being paid to the educational level and relationship status of our given population.

## Data Availability Statement

Data files can be accessed at https://figshare.com/s/bc18d5417d965c15a283.

## Ethics Statement

Expedited ethical approval from Kampala International Ethical Review Board was acquired and registered as Nr.UGREC-023/201914. An introductory letter was also obtained from community heads to facilitate community entry, especially with the Covid-19 lockdown. The patients/participants provided their written informed consent to participate in this study.

## Author Contributions

All authors reviewed the manuscript for intellectual content and approved the final version for publication, and remain in agreement to ensure that questions related to the integrity of any part of the work are resolved.

## Funding

This work was supported by Zhejiang University Education Foundation Emergency Research Fund (SCW and KIK); Global Challenges Research Fund and the University of Edinburgh.

## Conflict of Interest

The authors declare that the research was conducted in the absence of any commercial or financial relationships that could be construed as a potential conflict of interest.

## Publisher's Note

All claims expressed in this article are solely those of the authors and do not necessarily represent those of their affiliated organizations, or those of the publisher, the editors and the reviewers. Any product that may be evaluated in this article, or claim that may be made by its manufacturer, is not guaranteed or endorsed by the publisher.

## Supplementary Material

Supplementary file 1 The questionnaire.
