# Supplementary material for: COVID-19-Related Mental Health Burdens: Impact of Educational Level and Relationship Status Among Low-Income Earners of Western Uganda
Source: Front Public Health. 2021 Nov 18;9:739270. doi: 10.3389/fpubh.2021.739270 (PMC8663024; doi:10.3389/fpubh.2021.739270)
Supplement: Supplementary file 1 — The questionnaire. [file Data_Sheet_1.docx]

**Supplementary file 1:** COVID-19 Related Mental Health Burdens: Impact of Educational Level and Relationship Status among Low-Income Earners of Western Uganda

1. Sex

Male{ }

Female{ }

2. Age__________

3. Educational status

Primary level { }

Secondary level { }

Tertiary level { }

No formal education{ }

4. Marital status

Single{ }

Married{ }

Separated{ }

widowed{ }

**ASSESSMENT OF MENTAL HEALTH CARE AWARENESS**

5. Do you know what mental health care is?

Yes{ }

No{ }

6. Do you know any facility in Uganda where mental health care is provided?

Yes{ }

No{ }

7. Is there a place in your locality where mental health care is provided?

Yes{ }

No{ }

8. Do you have a pre-existing mental health challenge?

Yes{ }

No{ }

Not sure{ }

9. Which of the following mental health challenges applies to you?

Anxiety{ }

Depression{ }

Paranoia{ }

Anger issues{ }

Others{ }

Not available{ }

10. How do you handle your mental health challenge? (tick one or more)

Use of medication{ }

Drinking alcohol or smoking{ }

Watching movies{ }

Singing or dancing{ }

Binge eating{ }

others{ }

None of the above{ }

**ASSESSMENT OF ANXIETY USING MODIFIED GAD**

11. Have you heard of any mental health challenge related to Covid-19 before?

Yes{ }

No{ }

Maybe{ }

12. How has the Covid-19 updates and stories globally affect you? (tick one or more)

Makes me feel nervous or anxious{ }

Makes me worried{ }

Makes me restless or sleepless{ }

Makes me easily annoyed or irritable when some discusses it{ }

Makes me afraid I may become infected{ }

Indifferent { }

13. Since Covid-19 was confirmed in Uganda, how much have you adhered to Ministry of Health specified safety

obsessively{ }

moderately{ }

slightly{ }

not sure{ }

14. How do you feel if you unconsciously fail to observe or adhere to the ministry of health specified safety guidelines? (tick one or more)

Makes me feel nervous or anxious{ }

Makes me worried{ }

Makes me restless or sleepless{ }

Makes me easily annoyed or irritable when some discusses it{ }

Makes me afraid I may become infected{ }

Indifferent { }

15. Has the lockdown affected your source of income?

Yes{ }

No{ }

Prefer not to say{ }

16. If the lockdown has affected your source of income, how does that make you feel? (Tick one or more)

Makes me feel nervous or anxious{ }

Makes me worried{ }

Makes me restless or sleepless{ }

Makes me afraid that I may lose my job

Indifferent { }

**ASSESSMENT OF ANGER USING MODIFIED STAXI-2**

17. How does the lockdown make you feel? (tick one or more)

I feel Angry{ }

I feel furious{ }

I feel like hitting or kicking something

I feel irritated{ }

I feel annoyed{ }

I feel mad{ }

I feel like breaking things{ }

Indifferent { }

18. If you are a parent with young children, how does this lockdown with little ones make you feel? (tick one or more)

I feel Angry{ }

I feel furious{ }

I feel like hitting or kicking something

I feel irritated{ }

I feel annoyed{ }

I feel mad{ }

I feel like breaking things{ }

Not applicable{ }

19. Has the lockdown made the young children staying with you to spoil or break any household items and how does that make you feel? (tick one or more)

I feel Angry{ }

I feel furious{ }

I feel like hitting or kicking something

I feel irritated{ }

I feel annoyed{ }

I feel mad{ }

I feel like breaking things{ }

Not applicable{ }

20. Have you been spending more on feeding during the lockdown?

Yes{ }

No{ }

Not sure{ }

21. If yes, how does that make you feel? (tick one or more)

I feel Angry{ }

I feel furious{ }

I feel like hitting or kicking something

I feel irritated{ }

I feel annoyed{ }

I feel mad{ }

I feel like breaking things{ }

22. Are you worried about your finances or foodstuffs finishing during the lockdown?

Yes{ }

No{ }

Not sure{ }

23. If yes, how does that make you feel? (tick one or more)

I feel Angry{ }

I feel furious{ }

I feel like hitting or kicking something

I feel irritated{ }

I feel annoyed{ }

I feel mad{ }

I feel like breaking things{ }

Indifferent { }

**ASSESSMENT OF DEPRESSION USING MODIFIED BDI**

24. How do you feel about your sleeping pattern since the lockdown began?

I do not feel sad about it{ }

I feel sad about it{ }

I am sad all the time and can’t snap out of it{ }

I am so sad and unhappy about it

Indifferent { }

25. Has this lockdown affected your weight?

I haven’t lost much weight if any{ }

I have lost more than 2.5kg{ }

I have lost more than 5kg{ }

I have lost more than 7.5kg{ }

Not sure{ }

26. If you have gained more weight, how does that make you feel?

I do not feel sad{ }

I feel Sad{ }

I feel unhappy{ }

I feel annoyed{ }

Indifferent { }

27. How has the lockdown affected your zeal towards socializing?

I have not lost interest in socializing with people{ }

I am less interested in socializing with people than I used to be{ }

I have lost most of my interest in socializing with other people{ }

I have lost all my interest in socializing with other people{ }

Did not affect if{ }

28. What has been your coping mechanism to maintain your mental health during the lockdown? (Tick one or more)

Watching movies{ }

Reading { }

Engaging in meditation{ }

Personal development in form of learning new skills{ }

Eating more than I used to{ }

Others{ }

29. How satisfied are you with what you earn monthly from your work or business?

I do not feel like a failure{ }

I feel like I have failed myself{ }

I wished I could get a better job or pay{ }

I feel I am a complete failure as a person

Indifferent { }

30. If covid-19 doesn’t have a cure or vaccine, how do you feel about the future?

I am not particularly discouraged about the future{ }

I feel discouraged about the future{ }

I feel I have nothing to look forward to

I feel the future is hopeless and that things cannot change { }

**THANK YOU**
